# miR-3191 promotes the proliferation and metastasis of hepatocellular carcinoma via regulating PAK6

**DOI:** 10.1186/s13027-024-00628-w

**Published:** 2024-12-18

**Authors:** Anqi Xie, Hengjie Wang, Jingchen Huang, Minmin Sun, Lin Chen

**Affiliations:** 1 Community Health Service Center, Zhongshan Street, Songjiang District, Shanghai, China; 2 Department of Hepatic Surgery, Kunshan Hospital of Traditional Chinese Medicine, Kunshan, Jiangsu Province 215300 China; 3 The National University of Malaysia, Kuala Lumpur, Malaysia; 4 Department of Hepatic Surgery, Third Affiliated Hospital of Second Military Medical University, 225 Changhai Road, Shanghai, 200438 China

**Keywords:** Hepatocellular carcinoma, miR-3191, p21-activated protein kinase 6, Prognosis, Progression

## Abstract

**Background/Aims:**

microRNAs (miRNAs) contribute to tumorigenesis, progression and drug resistance of hepatocellular carcinoma (HCC). miR-3191 is a newly discovered miRNA, and its function and mechanism of action in biological processes and diseases are not completely understood.

**Methods:**

miR-3191 expression is determined via quantitative real-time polymerase chain reaction. Knockdown and overexpression of miR-3191 influence the proliferation and metastasis of HCC cells, which is measured by Cell Counting Kit-8 assay, Colony Formation assay and Cell metastasis assay. Protein expression is estimated by Western blot. The interplay between miR-3191 and target is validated by dual-luciferase reporter assay.

**Results:**

Here, we show that miR-3191 is upregulated in HCC tissues and associated with poor prognosis of HCC patients. Mechanistically, p21-activated protein kinase 6 (PAK6) was identified as a direct target of miR‑3191 in HCC. PAK6 knockdown partially recovered interference of miR‑3191‑induced decrease in cell proliferation and invasion. The accuracy of HCC patient prognosis could be improved by employing a combination of miR-3191 and PAK6 values.

**Conclusions:**

miR-3191 promotes the proliferation and metastasis of HCC cells via targeting PAK6 and may serve as a prognostic biomarker and potential therapeutic target.

**Supplementary Information:**

The online version contains supplementary material available at 10.1186/s13027-024-00628-w.

## Introduction

Hepatocellular carcinoma (HCC) ranks the sixth most common form of cancer worldwide, with about 870,000 cases reported in 2022 [1]. Hepatitis B virus (HBV), poor diet and inactivity, fungal toxins, non-alcoholic fatty liver disease (NAFLD), alcohol consumption and infection with the hepatitis C virus are major pathogenic factors for HCC [2–5]. Due to the unobvious early symptoms, most HCC patients are diagnosed at advanced stage. Surgical resection is the first choice for most patients, while postoperative recurrence and metastasis rates are particularly high [6, 7]. Despite the great advances achieved in chemotherapy, targeted therapy and immunotherapy, patients with HCC generally have a poor prognosis [8, 9]. Therefore, it’s urgent to explore the underlying molecular pathogenesis of HCC and to develop effective intervention strategies.

MicroRNAs (miRNAs) are single-stranded small RNA molecules about 21–23 bases in size, which are generated by the dicer enzyme processing of single-stranded RNA precursors about 70–90 bases in size with hairpin structure [10]. Lin-4 and let-7 were the first identified miRNAs, which were found in nematode worms [11]. Subsequently, several research groups identified hundreds of miRNAs in a variety of biological species, including humans, fruit flies, plants and so on [12, 13]. MiRNAs have high biological genome coding and degrades mRNA or blocks its translation by pairing with target gene mRNA base to guide silencing complex (RISC). Increasing evidence found that miRNAs play a variety of roles in the regulation of cell growth and development. In addition, numerous studies showed that miRNAs participated in the regulation of tumorigenesis, progression and drug resistance of human malignant tumors [14–17]. miR-3191 is a newly discovered miRNA, and its function and mechanism of action in biological processes and diseases are not completely understood [18]. It was reported that miR-3191 promotes migration and invasion by downregulating TGFBR2 in colorectal cancer [19]. Nonetheless, the role of miR‑3191 and its underlying mechanism in the progression of HCC remain poorly understood.

In this study, we for first find that miR-3191 is highly expressed in HCC tissues and predicts poor prognosis of HCC patients. Next, by using loss-of-function analysis and gain-of-function analysis in HCC cells, we demonstrate that miR-3191 promotes HCC cells proliferation and metastasis. Further mechanism study reveals that PAK6 is a direct target of miR-3191 in HCC cells. Clinical investigation also confirms the correlation between miR-3191 and PAK6, and demonstrates the value of combining miR-3191 and PAK6 to improve the prognostic accuracy for HCC patients. Altogether, we discover that miR-3191 promotes the proliferation and metastasis of HCC cells via targeting PAK6.

### Materials and methods

### Patients and samples

HCC samples were collected from patients who underwent the resection of their primary HCC in Eastern Hepatobiliary Surgery Hospital (EHBH, Shanghai, China). Cohort 1 patients (*n* = 180, from 2009 to 2013) and cohort 2 patients (*n* = 113, from 2011 to 2014) were collected to investigate the clinical significance of miR-3191 in HCC. Detailed clinicopathological features are described in supplementary Tables 1&2. Overall survival (OS) was defined as the interval between the dates of surgery and death. The recurrence was defined as the interval between the dates of surgery and recurrence; if recurrence was not diagnosed, then patients were censored on the date of death or on the last follow-up. Forty human HCC tissues and normal tissues, Eighteen paired peri-tumor normal tissue, primary HCC and metastatic foci, Sixteen paired primary HCC and recurrent HCC tissues, and Sixty-three differentially differentiated human HCC tissues were collected to detect miR-3191 expression. The patients’ informed consent was also obtained, and all procedures were approved by the ethical committee of EHBH.

### Cell lines and cell culture

HCC cell lines Hep3B and Huh7 were provided by Erasmus University (Rotterdam, Netherlands). All human HCC cells were cultured in DMEM (Gibco^®^, New York, USA) with 10% FBS (Gibco^®^), glutamine and penicillin/streptomycin (Gibco^®^) at 37 °C in 5% CO_2_ incubator. Mycoplasma contamination was excluded via a PCR (D7232, Beyotime, Shanghai, China) based method. The cell lines were authenticated by short tandem repeat (STR) DNA profiling.

The lenti-vector expressing miR-3191 mimic, miR-3191 inhibitor and their control virus were purchased from Shanghai GenePharma (Shanghai, China). NC mimic: 5’-UUCUCCGAACGUGUCACGUTT-3’, miR-3191 mimic: 5’-UGGGGACGUAGCUGGCCAGACAG-3’, NC inhibitors: 5’-CAGUACUUUUGUGUAGUACAA-3’, miR‐3191 inhibitors: 5’-CUGUCUGGCCAGCUACGUCCCCA-3’. siPAK6 and its control siRNA were purchased from Shanghai GenePharma. Huh7 and Hep3B cells were infected with miR-3191 mimic, miR-3191 inhibitor and their control virus and the stable infectants were screened by puromycin as described previously [20].

### Cell counting kit-8 (CCK-8) assay

CCK-8 assay was performed according to the manufacturer’s instructions to measure cell proliferation. Then, 3 × 10^3^ HCC cells were plated and incubated in 96-well plates for 0, 24, 48, 72 and 96 h. They were placed in an incubator containing 5% CO_2_ at 37 °C. Next, 10 µL of CCK-8 reagent was added to each well for 1 h (Dojindo Molecular Technologies, Kumamoto, Japan). Finally, they were detected using a microplate reader (Molecular Devices, Kumamoto, Japan) at an absorbance of 450 nm.

### Colony formation assay

For colony formation assay, HCC cells were cultured in 96-well plates with 1,000 cells/well at 37 °C for 7 days, and were subsequently stained with 0.1% crystal violet (C0121, Beyotime, Shanghai, China). Then, a light microscope (ThermoFisher Scientific, USA) was used to count the colonies.

### 5-ethynyl-2’-deoxyuridine (EdU) immunofluorescence staining

For cell EdU immunofluorescence staining, HCC cells were seeded into 96-well plates (3 × 10^3^ cells) and performed using the EdU Kit (C0071S, Beyotime, Shanghai, China). The results were quantified with a Zeiss axiophot photomicroscope (Carl Zeiss) and Image-Pro plus 6.0 software.

### Cell metastasis assays

For cell migration experiments, 2 × 10^5^ HCC cells were seeded into the upper chamber of a polycarbonate transwell in serum-free DMEM medium. The lower chamber was added with DMEM medium containing 20% FBS as chemoattractant. The cells were incubating for 16 h and the chamber was fixed with 10% neutral formalin for more than 4 h. The cells were dyed with crystal violet. The cells were then counted under a microscope (Olympus) and the cell number is expressed as the average number of the cells in each field.

For cell invasion experiments, 2 × 10^5^ HCC cells were seeded into the upper chamber of a polycarbonate transwell in serum-free DMEM medium. The lower chamber was added with DMEM medium containing 20% FBS as chemoattractant. The cells were incubating for 36 h and the chamber was fixed with 10% neutral formalin for more than 4 h. The cells were dyed with crystal violet. The cells were then counted under a microscope (Olympus) and the cell number is expressed as the average number of the cells in each field.

### Animal models

BALB/c nude mice (male, 6 weeks old) were purchased from Chinese Academy of Sciences Slack Company (Shanghai, China). For xenograft formation assay, 2 × 10^6^ Huh7 miR-3191 inhibitor cells and control cells were injected subcutaneously into nude mice (*n* = 6 each, randomized allocated). Nude mice were sacrificed six weeks post inoculation and tumors were collected and xenografts weight examined.

For lung metastatic model, a total of 2 × 10^6^ Huh7 miR-3191 inhibitor cells and control cells were injected into the tail veins of nude mice (*n* = 6 each, randomized allocated). Mice were sacrificed at 2 months post injection. The lung tissues of each mouse were separated and subjected to H&E staining. And the lung metastatic foci were counted in a double-blind manner with the aid of a dissecting microscope. All animal experiments were approved by the Animal Care Committee of Community Health Service Center.

### Real-time PCR

Real-time PCR were carried out as described in our previous paper [20]. Briefly, total RNA was extracted using an miRcute miRNA isolation kit (TIANGEN, Beijing, China) for miRNA and TRIzol (Life Technologies, USA) for mRNA in accordance with the manufacturer’s instructions. Then, miRNA and mRNA were polyadenylated using a poly-A polymerasebased First-Strand Synthesis kit (Takara, Kyoto, Japan). Subsequently, a PrimeScript RT Reagent kit (TaKaRa) were used for reverse transcription (RT) of the total mRNA. Finally, miR-3191 and complementary DNA (cDNA) in these samples were assayed using SYBR Green I (Applied Biosystems, USA). The primer sequences were: PAK6 primer sequences were forward: 5’ GCTCTCGGACTTCGGATTCT 3’, reverse: 5’ GGCATACAAAGACCTGGAGAT 3’. The β-actin was used as reference for relative expression calculation and its primer sequences were forward: 5’ GGCCCAGAATGCAGTTCGCCTT 3’, reverse: 5’ AATGGCACCCTGCTCACGCA 3’.

### Western blotting assay

Protein extraction from HCC cells were finished by using the RIPA lysis buffer reagent (P0013B, Beyotime, Shanghai, China), and the protein quality was determined by BCA kit (P0009, Beyotime). Next, proteins were separated by SDS-PAGE, transferred onto the PVDF membranes (Millipore, USA), and incubated with primary antibodies against PAK6, N-cadherin, E-cadherin, and Vimentin, and subsequently with the secondary antibodies. Finally, an electrochemiluminescence (ECL) system (5200CE, Tanon, Shanghai, China) was used for protein bands visualization, which were analyzed by using the Image J software. The primary antibodies were E-cadherin (1:1000; 20874-1-AP, Proteintech), N-cadherin (1:1000; 22018-1-AP, Proteintech), PAK6 (1:1000; 13539-1-AP, Proteintech) and GAPDH (1:5000; #5174, Cell Signaling Technology).

### Luciferase reporter assay

The 3’-untranslated region (3’-UTR) of wildtype (GUCCCCA) or mutant (ACAGUAC) PAK6 was inserted into the pmir-GLO Luciferase vector (Promega, Madison, WI, USA) for Luciferase reporter experiments. Then, the above Luciferase plasmids were transfected into miR-3191 mimic or inhibitor and their control HCC cells. Finally, cells were extracted with passive lysis buffer [25 mM Tris-phosphate (pH 7.8), 2 mM EDTA, 1% Triton X-100, and 10% glycerol]. The luciferase activity was measured with a microplate luminometer LB 96 V (Berthold GmbH & Co. KG, Bad Wildbad, Germany). The Renilla luciferase signal was normalized to the internal firefly luciferase transfection control.

### RNA immunoprecipitation (RIP) assay

The RIP assay were used to verify the binding relationship between miR-3191 and PAK6. The RIP assay was performed using the Magna RIP™ RNA Binding Protein Immunoprecipitation Kit (Millipore, USA) according to the manufacturer’s instructions. Cells were lysed in RIP lysis buffer, and then were incubated with RIP buffer with a human anti-Ago2 antibody (Abcam, Cambridge, MA, USA). Each sample was incubated with proteinase K to digest protein. Purify RNA was obtained and then was analyzed using qRT-PCR assay.

### Immunohistochemistry

The tissue samples were fixed with 10% neutral formaldehyde, embedded in paraffin, and sectioned for immunohistochemical (IHC) staining. In brief, after antigen retrieval, sections or tissue microarrays (TMAs) were blocked with bovine serum antigen albumin (BSA) and incubated with the indicated primary antibody and then secondary antibody. A diaminobenzidine (DAB) colorimetric reagent solution was used, followed by hematoxylin counterstaining. The slides were scanned, and representative images were captured. IHC scoring was based on the percentage of positively stained cells, and staining intensity was assessed by Image Scope software (Aperio Technologies, Inc.).

### Statistical analysis

Each experiment was repeated at least three times. Statistical analysis was performed using SPSS V.18.0. The data are expressed as the mean ± SD. Two-tailed Student’s t test or the Mann-Whitney U test was used to compare two continuous variables, and the chi-square test was used to compare qualitative variables. The DFS and OS of distinct subgroups were compared by the Kaplan-Meier method and log-rank analysis. Pearson’s correlation analysis was performed to determine the correlation between two variables. Each dataset was analyzed separately. A p value less than 0.05 was considered statistically significant.

## Results

### miR-3191 expression is upregulated in HCC tissues

**Fig. 1:**
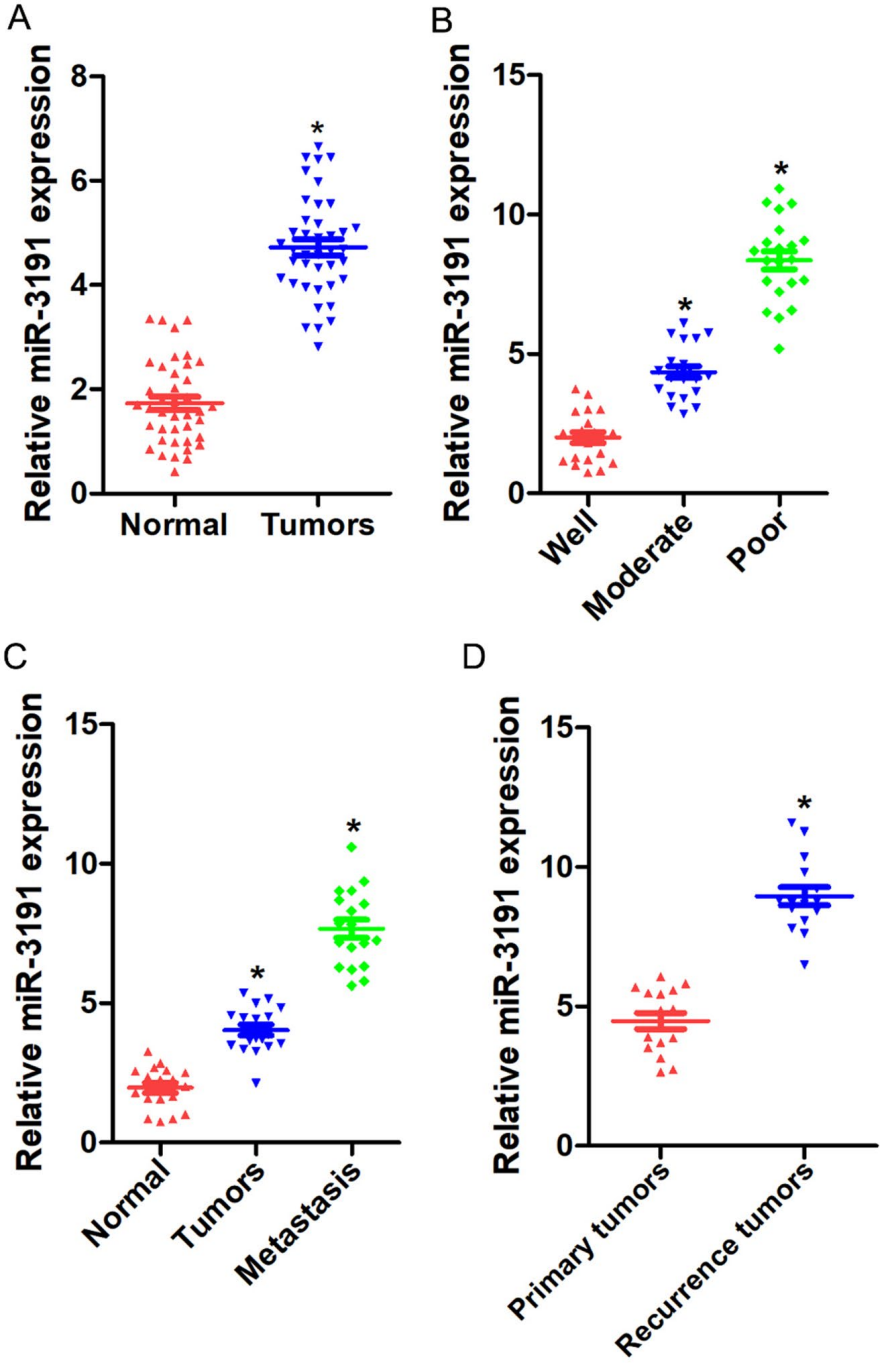
Up-regulation of miR-3191 in HCC tissue (**A**) miR‑3191 expression in 40 pairs of HCCs and peri-tumor normal tissues. (**B**) miR‑3191 expression in 63 differentially differentiated human HCCs. (**C**) miR‑3191 expression in 18 paired peri-tumor normal tissue, primary HCC and metastatic foci. (**D**) miR‑3191 expression in 16 primary HCC and recurrent HCC tissues

To explore the potential role of miR-3191 in HCC, we examined miR-3191 expression levels in a large number of human HCCs. As shown in Fig. 1A, miR-3191 expression was significantly increased in HCC tissues compared with the paired non-cancerous tissues. Consistently, poorly differentiated HCCs exhibited higher expression levels of miR-3191, while relatively lower miR-3191 amounts were detected in those well-differentiated HCCs (Fig. 1B). Existing evidence showed that HCC carries a high risk of portal vein metastasis and recurrence [21]. Strikingly, miR-3191 expression was dramatically elevated in metastatic foci compared with the matched primary HCCs or peri-tumor normal tissues (Fig. 1C), which indicates the potential function of miR-3191 in HCC metastasis. Moreover, miR-3191 expression was markedly increased in recurrent HCC foci compared with the matched primary HCCs (Fig. 1D).

### miR-3191 predicts the poor prognosis of HCC patients

**Fig. 2:**
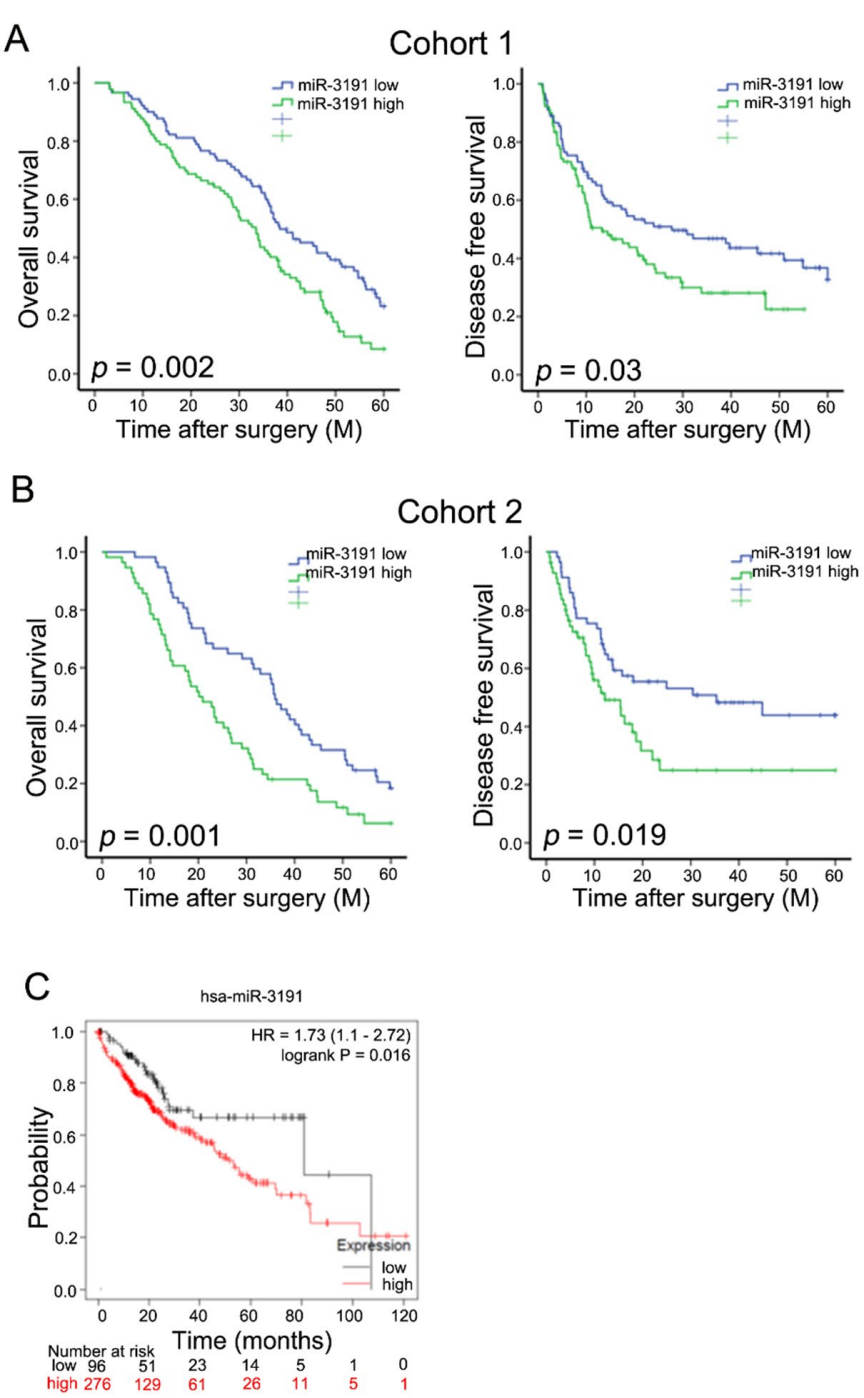
miR-3191 predicts the poor prognosis of HCC patients (**A**) Overall survival and disease free survival time after surgery of the 180 HCC patients cohort were compared between the ‘‘miR-3191 low’’ (*n* = 90) and ‘‘miR-3191 high’’ (*n* = 90) groups, *p* < 0.05. (**B**) Overall survival and disease free survival time after surgery of the 113 HCC patients cohort were compared between the ‘‘miR-3191 low’’ (*n* = 57) and ‘‘miR-3191 high’’ (*n* = 56) groups, *p* < 0.05. (**C**) Kaplan-Meier survival curves of OS based on miR-3191 expression in HCC using the online bioinformatics tool Kaplan-Meier plotter

To investigate the clinical significance of miR-3191, we used two independent HCC cohorts. Notably, correlation regression analysis revealed that high miR-3191 expression in HCC tissues was associated with aggressive clinical features (Supplementary Tables 1 and 2). Moreover, patients with higher miR-3191 levels exhibited worse overall survival and shorter time to recurrence (Fig. 2A&B). Moreover, using the online bioinformatics tool Kaplan-Meier plotter, we found that HCC patients with increased miR-3191 expression had worse overall survival (Fig. 2C). Thus, miR-3191 expression may serve as a valuable predicting factor for the survival of HCC patients.

### miR-3191 knockdown inhibits cell proliferation and metastasis in HCC

**Fig. 3:**
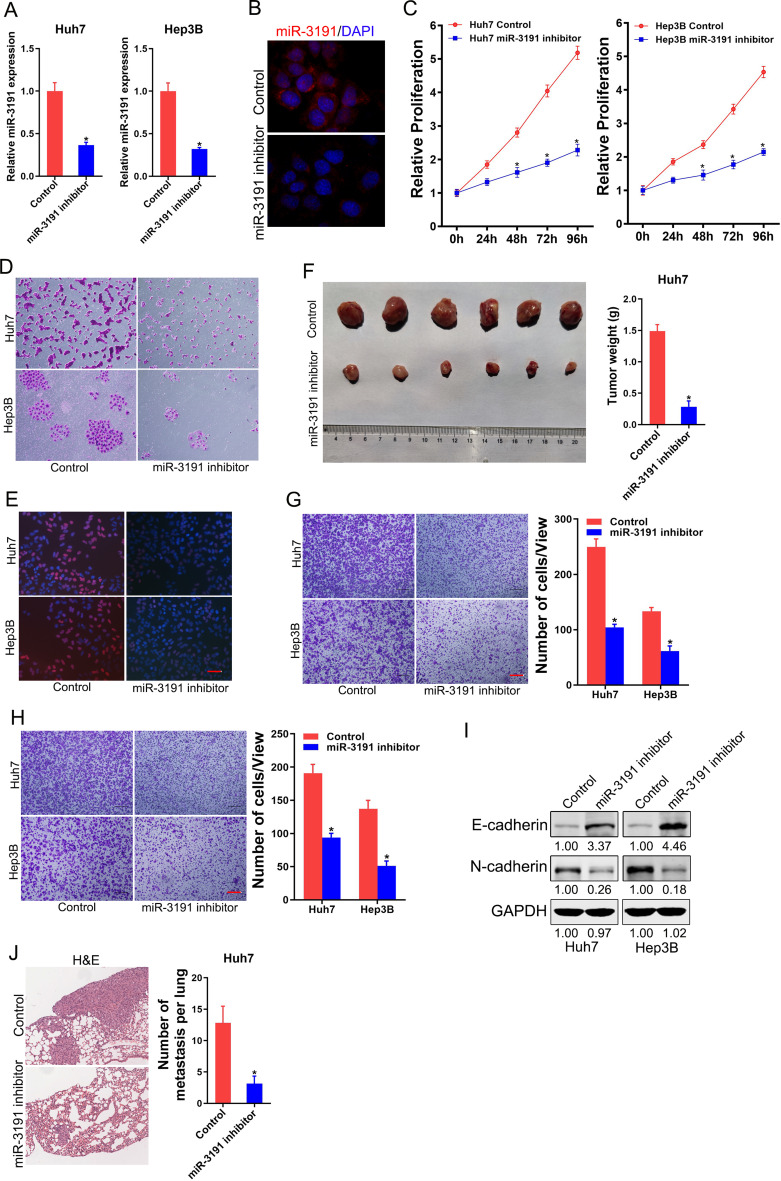
miR-3191 knockdown inhibits HCC cells proliferation and metastasis (**A**) Relative expression of miR-3191 in Huh7 and Hep3B cells after infection of miR-3191 knockdown virus and control virus. (**B**) FISH analysis of miR-3191 in Huh7 miR‑3191 inhibitor and control cells using biotin-labelled LNA probe. The nuclei were stained with DAPI. (**C**) Relative proliferation curve of Huh7 and Hep3B treated with miR-3191 inhibitor and control cells at 0, 24, 48, 72 and 96 h (*n* = 4). (**D**) The proliferation of Huh7 and Hep3B treated with miR-3191 inhibitor and control cells were assessed using cell colony formation (*n* = 3). (**E**) EdU assay of Huh7/Hep3B miR‑3191 inhibitor and control cells (*n* = 3). Scale bar = 25 μm. (**F**) 2 × 10^6^ Huh7 miR-3191 inhibitor and control cells were subcutaneously inoculated into nude mice (*n* = 6) and excised six weeks later. The weight of xenografted tumors from different groups was compared. (**G**) Transwell migration of Huh7 and Hep3B treated with miR-3191 inhibitor and control cells (*n* = 3). Scale bar = 100 μm. (**H**) Transwell invasion of Huh7 and Hep3B treated with miR-3191 inhibitor and control cells (*n* = 3). Scale bar = 100 μm. (**I**) Western blot analysis of E-cadherin and N-cadherin protein expression in Huh7 and Hep3B treated with miR-3191 inhibitor and control cells. (**J**) Lung H&E staining of nude mice inoculated Huh7 miR-3191 inhibitor or control cells via tail vein for 12 weeks. The number of lung metastatic foci in each group (*n* = 6) were also calculated, *p < 0.05. Scale bar = 200 μm

Next, miR-3191 knockdown virus was infected into Huh7 and Hep3B cells to investigate its role in HCC. Real-time PCR assay showed that miR-3191 expression was much lower in miR-3191 inhibitor Huh7 and Hep3B cells than control HCC cells (Fig. 3A). Moreover, fluorescence in situ hybridization (FISH) assay also showed that miR-3191 expression was much lower in miR-3191 inhibitor Huh7 cells than control Huh7 cells (Fig. 3B). CCK-8 assay showed that knockdown of miR-3191 inhibited cells proliferation in HCC (Fig. 3C). Moreover, the colony assay demonstrated that the HCC cells with miR-3191 knockdown virus developed fewer and smaller colonies (Fig. 3D). EdU staining assay further indicated that interference of miR-3191 attenuated HCC cells proliferation (Fig. 3E). Importantly, miR-3191 knockdown HCC cells formed much smaller xenografts in vivo (Fig. 3F). To determine the effects of miR‑3191 on the migration and invasion of HCC cells, the Transwell chamber assay was performed. As expected, miR‑3191 knockdown significantly impaired the migratory and invasive abilities of both Huh7 and Hep3B cells (Fig. 3G&H). In addition, downregulation of miR-3191 significantly increased expression of epithelial marker (E-cadherin) and decreased expression of mesenchymal marker (N-cadherin) (Fig. 3I). Moreover, miR-3191 knockdown HCC cells formed decreased numbers of pulmonary lesions in mice (Fig. 3J).

### miR-3191 promotes HCC cell proliferation and metastasis

**Fig. 4:**
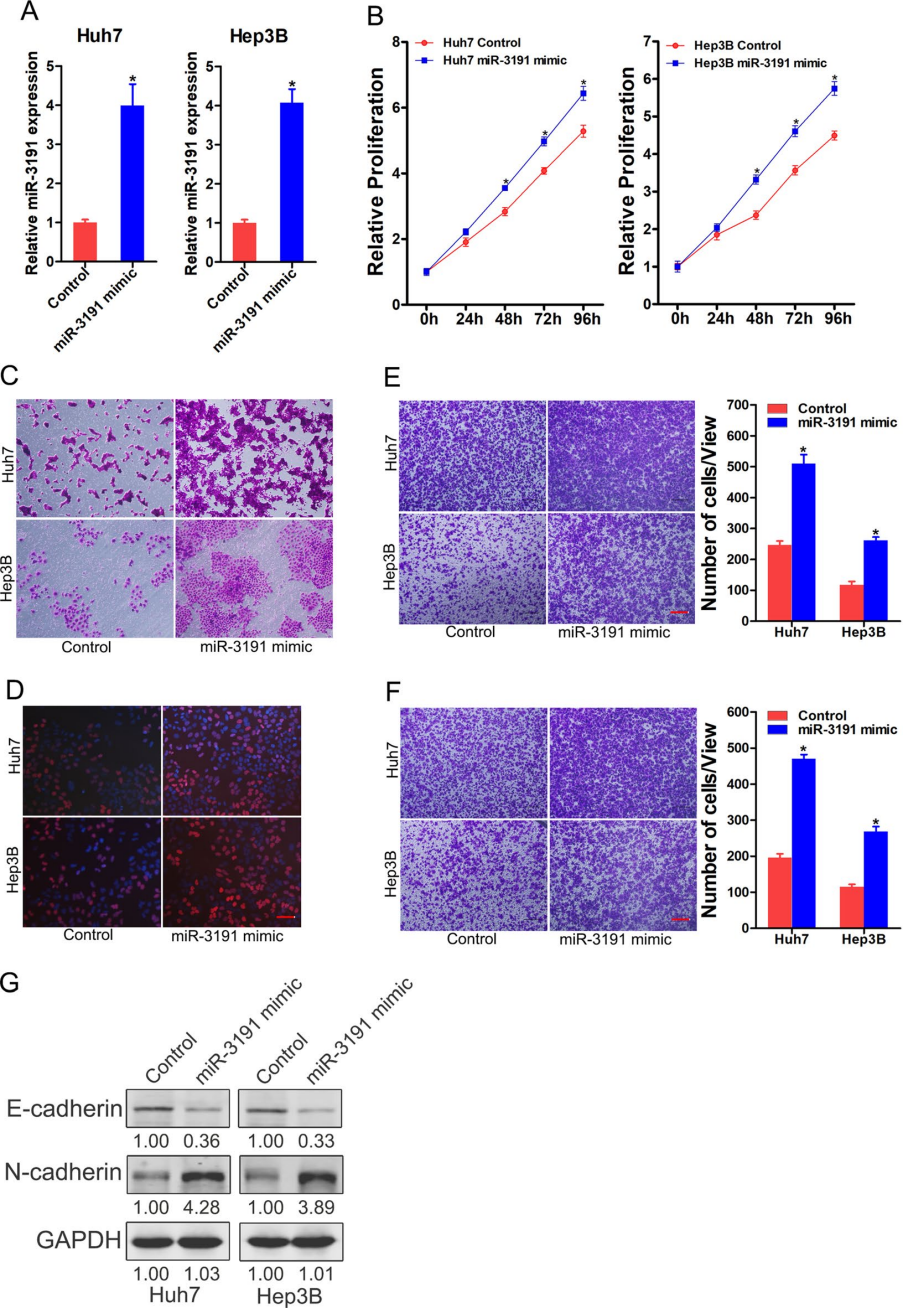
miR-3191 promotes HCC cells proliferation and metastasis (**A**) Relative expression of miR-3191 in Huh7 and Hep3B cells after infection of miR-33,191 overexpression virus or control virus (*n* = 3). (**B**) Relative proliferation curve of Huh7 and Hep3B treated with miR-3191 mimic and control cells at 0, 24, 48, 72 and 96 h (*n* = 4). (**C**) Relative proliferation of Huh7 and Hep3B treated with miR-3191 mimic and control cells (*n* = 3). (**D**) EdU assay of Huh7/Hep3B miR‑3191 mimic and control cells (*n* = 3). Scale bar = 25 μm. (**E**) Transwell migration of Huh7 and Hep3B treated with miR-3191 mimic and control cells (*n* = 3). Scale bar = 100 μm. (**F**) Transwell invasion of Huh7 and Hep3B treated with miR-3191 mimic and control cells (*n* = 3). Scale bar = 100 μm. (**G**) Western bolt analysis of E-cadherin and N-cadherin protein expression in Huh7 and Hep3B treated with miR-3191 mimic and control cells

To further explore its role in HCC, miR-3191 overexpression virus was infected into Huh7 and Hep3B cells. Real-time PCR assay showed that miR-3191 expression was much higher in miR-3191 mimic Huh7 and Hep3B cells than control HCC cells (Fig. 4A). CCK-8 assay showed that overexpressing of miR-3191 promoted cells proliferation in HCC (Fig. 4B). Moreover, the colony assay demonstrated that the miR-3191 overexpression HCC cells formed much more and bigger colonies (Fig. 4C). EdU staining assay further indicated that overexpressing of miR-3191 facilitated HCC cells proliferation (Fig. 4D). Furthermore, forced miR‑3191 expression dramatically enhanced the migratory and invasive abilities of both Huh7 and Hep3B cells (Fig. 4E&F). Moreover, up-regulation of miR-3191 significantly decreased expression of epithelial marker (E-cadherin) and increased expression of mesenchymal marker (N-cadherin) (Fig. 4G).

### miR-3191 promotes HCC cells proliferation and metastasis via directly targets PAK6

**Fig. 5:**
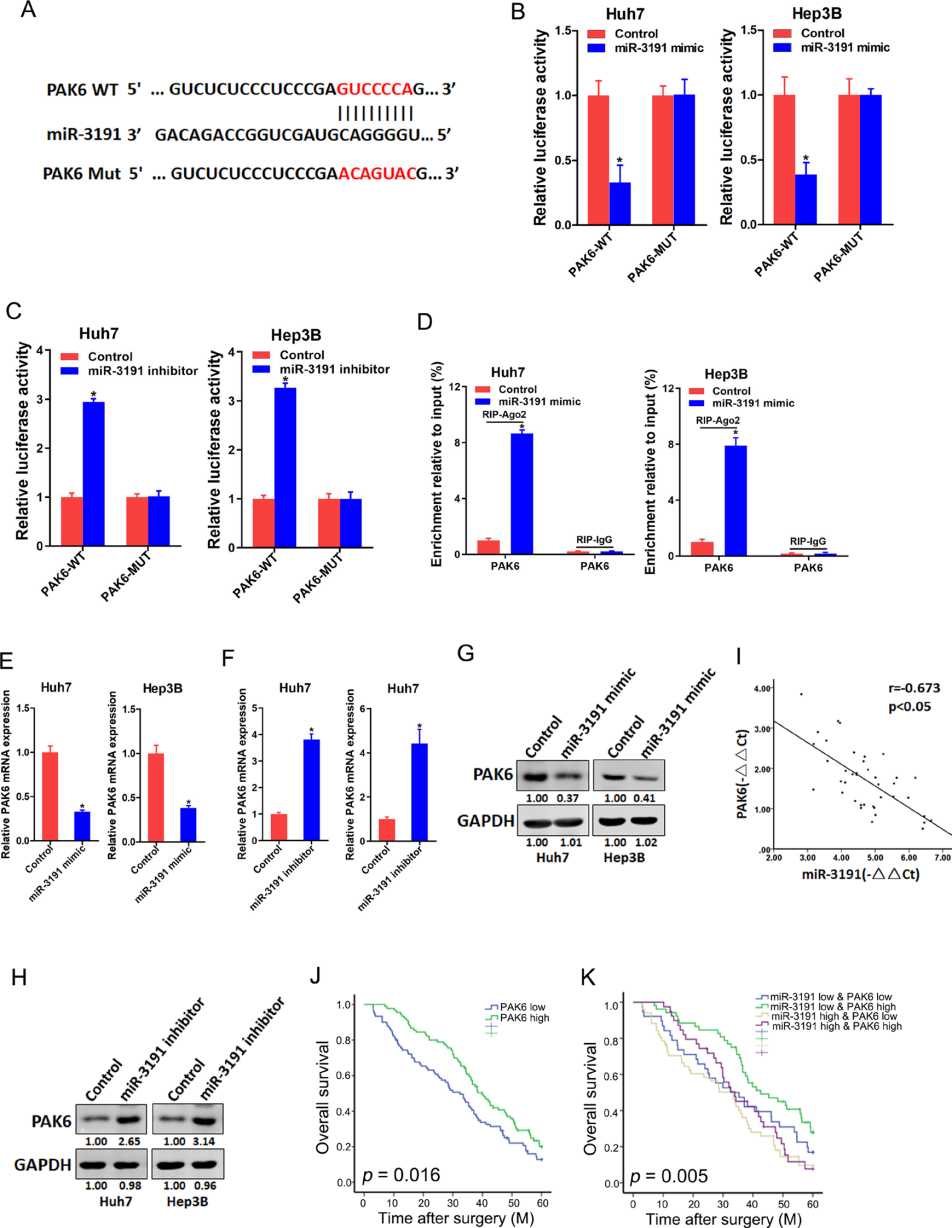
PAK6 is a directly target of miR-3191 in HCC cells (**A**) A potential target site for miR-3191 in the 3’-UTR of human PAK6 mRNA, as predicted by the program TargetScan. To disrupt the interaction between miR-3191 and PAK6 mRNA, the target site was mutated. (**B**) Luciferase activity of PAK6‑3’‑UTR‑WT or PAK6‑3’‑UTR‑MUT plasmids in Huh7/Hep3B miR‑3191 mimic and control cells (*n* = 3). (**C**) Luciferase activity of PAK6‑3’‑UTR‑WT or PAK6‑3’‑UTR‑MUT plasmids in Huh7/Hep3B miR‑3191 inhibitor and control cells (*n* = 3). (**D**) Expression of PAK6 in Huh7 and Hep3B cells treated with miR-3191 mimic and control. (**E**) Relative PAK6 mRNA expression in Huh7 and Hep3B treated with miR-3191 mimic and control cells (*n* = 3). (**F**) Western blot analysis of PAK6 protein expression in Huh7 and Hep3B treated with miR-3191 mimic and control cells. (**G**) PAK6 mRNA expression in Huh7 and Hep3B treated with miR-3191 inhibitor and control cells (*n* = 3). (**H**) PAK6 protein expression in Huh7 and Hep3B treated with miR-3191 inhibitor and control cells. (**I**) Significant correlation between miR-3191 and PAK6 expression in human HCC tissues (*n* = 40). (**J**) Overall survival time after surgery of the patients were compared between the ‘‘PAK6 low’’ (*n* = 90) and ‘‘PAK6 high’’ (*n* = 90) groups, *p* < 0.05. (**K**) Kaplan–Meier analysis of overall survival of TNBC patients with high or low miR-3191 and PAK6. *p* < 0.05

In probing the underlying mechanism, the online TargetScan software (http://www.targetscan.org/vert_72/) was used to predict the possible target genes of miR-3191 in HCC. The results showed that miR-3191 potentially bound to the 3’-UTR of PAK6 mRNA (Fig. 5A). To investigate this issue, the targeting sites in PAK6 were mutated, and were transfected into miR-3191 mimic or inhibitor and their control HCC cells, respectively. The decreased the luciferase activity mediated by miR-3191 overexpression and the increased the luciferase activity mediated by miR-3191 knockdown can be prevented through mutation of miR-3191 binding sites within the PAK6 mRNA 3’-UTR region (Fig. 5B&C). RIP assay showed that the endogenous PAK6 was significantly enriched in HCC cells co-transfected miR-3191 and PAK6 (Fig. 5D), revealing the direct binding between PAK6 and miR-3191.

Next, the PAK6 mRNA and protein expression was increased in miR-3191 knockdown HCC cells, while the opposite effect was observed when miR-3191 was overexpressed (Fig. 5E-H). Moreover, there was a negative correlation between miR-3191 and PAK6 mRNA in HCC tissues (Fig. 5I). Although either high miR-3191 or low PAK6 in HCC predicts a poor prognosis (Fig. 5J), HCC patients with both elevated miR-3191 level and reduced PAK6 expression displayed even worse prognosis (Fig. 5K), indicating a better prognostic value of combining the two parameters in comparison with miR-3191 or PAK6 alone.

**Fig. 6:**
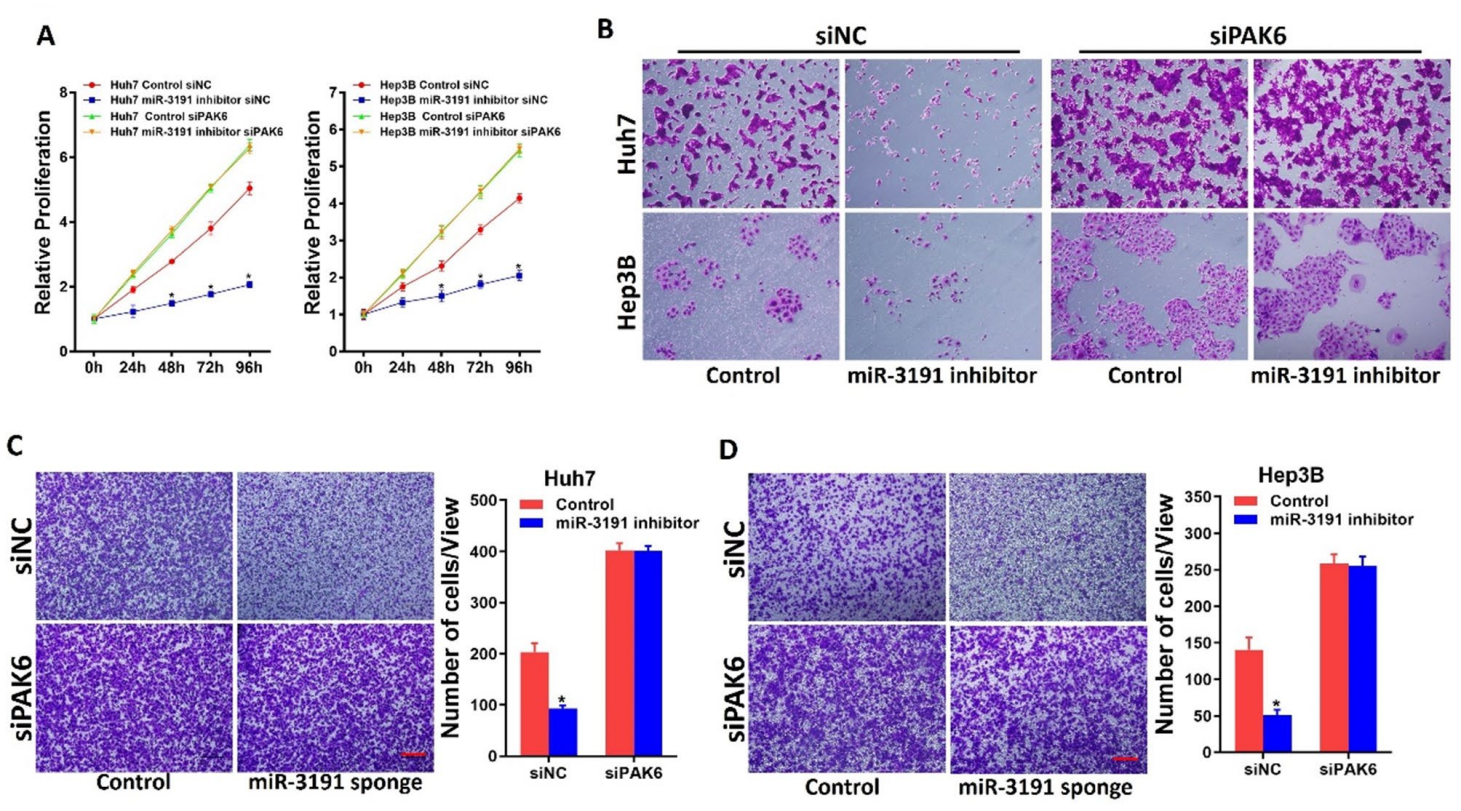
miR-3191 promotes HCC cells progression via targeting PAK6 (**A**) Relative proliferation of Huh7 and Hep3B treated with miR-3191 inhibitor and control cells and transfected with siPAK6 (*n* = 3). (**B**) Colony formation of Huh7 and Hep3B treated with miR-3191 inhibitor and control cells and transfected with siPAK6 (*n* = 3). (**C**&**D**) Invasion of Huh7 and Hep3B treated with miR-3191 inhibitor and control cells and transfected with siPAK6 (*n* = 3). Scale bar = 100 μm

To further elucidate whether the effects of miR‑3191 were mediated by repression of PAK6 in HCC cells, special PAK6 siRNA was used. As expected, the growth and metastasis ability in miR-3191 knockdown HCC cells could also be recovered through knockdown of PAK6 (Fig. 6A-D). Collectively, the above results demonstrated that miR-3191 promotes HCC cells proliferation and metastasis via targeting PAK6 signaling.

## Discussion

Despite the extensive investigation of miRNAs in HCC, there are few miRNAs can be used for clinical transformation. In this study, we identified miR-3191 as a novel HCC-related miRNA that was upregulated in HCC. Patient cohorts assay showed that high miR-3191 expression associated with poor prognosis of HCC patients. In vitro functional experiments revealed that miR-3191 significantly facilitated the proliferation, colony formation, migration and invasion of HCC cells. The underlying mechanism of miR-3191 function was associated the regulatory effect it had on PAK6 expression. Our study highlights the critical role of the miR-3191/PAK6 signaling axis in regulating the malignant behavior of HCC cells.

miR-3191 is a newly discovered miRNA, and its function and mechanism of action in biological processes and diseases are unclear [18]. He et al. have shown that miR-3191 was upregulated in colorectal cancer tissues and promoted metastasis of colorectal cancer via targeting TGFBR2 [19]. In the present study, we also showed that miR-3191 was upregulated in HCC tissues. In addition, our clinical investigations revealed the close correlation between miR-3191 levels with HCC progression and patient prognosis. Moreover, we confirmed that miR-3191 increased the HCC cell proliferation by a series of in vitro and in vivo assays. Additionally, we also found that miR-3191 promoted the migrative and invasive ability of HCC cell lines in vitro. This is a novelty of our work that has not been reported in previous studies.

p21-activated protein kinase 6 (PAK6) is a serine-threonine kinase belonging to the PAK family, which associated with many fundamental cellular processes in cancer including cell-cell adhesion, migration and apoptosis [22–24]. Previous studies have indicated that abnormal expression of PAK6 played critical roles in numerous types of cancers, including cervical cancer, prostate cancer, gastric cancer and liver cancer [25–27]. Chen and colleagues have reported that PAK6 overexpression is involved in the pathogenesis of HCC and may be an independent poor prognostic factor for HCC [28]. However, Liu and colleagues found that decreased PAK6 expression predicts poor survival in HCC patients [29]. In the present study, bioinformatics results showed that miR-3191 can bind to the 3’-UTR of PAK6. Forced miR-3191 expression downregulated PAK6 mRNA and protein in HCC cells. miR-3191 negatively correlated with PAK6 mRNA in HCC tissues. Moreover, PAK6 siRNA further confirm that miR-3191 promoted HCC proliferation and metastasis via regulating PAK6 pathway. Consistently, our results showed that either high miR-3191 expression or low PAK6 correlated with poor prognosis of HCC patients. Accumulating evidence indicates that appropriate combination of different markers might be more accurate than a single marker in prognosis evaluation. Herein, we reported that the combination of high miR-3191 expression and low PAK6 predicted worse prognosis than either marker alone, suggesting a more accurate combinational marker to evaluate the prognosis of HCC patients.

In summary, our study for the first time showed that miR-3191, as an oncogene, promoted HCC cells proliferation and metastasis through the translational repression of PAK6. miR-3191 might serve as a novel target for PAK6 prediction and therapy.

## Electronic supplementary material

Below is the link to the electronic supplementary material.

Supplementary Material 1

## Data Availability

The data in the current study are available from the corresponding authors upon reasonable request.

## References

[1] Bray F, et al. Global cancer statistics 2022: GLOBOCAN estimates of incidence and mortality worldwide for 36 cancers in 185 countries. CA Cancer J Clin. 2024;74(3):229–63. 38572751 10.3322/caac.21834

[2] Sung H et al. Global cancer statistics 2020: GLOBOCAN estimates of incidence and mortality worldwide for 36 cancers in 185 countries. CA Cancer J Clin, 2021. 10.3322/caac.21660 33538338

[3] Llovet JM, et al. Hepatocellular carcinoma. Nat Rev Dis Primers. 2016;2:16018. 27158749 10.1038/nrdp.2016.18

[4] Schuppan D, Afdhal NH. Liver cirrhosis. Lancet. 2008;371(9615):838–51. 18328931 10.1016/S0140-6736(08)60383-9 PMC2271178

[5] Huang DQ, et al. Global epidemiology of alcohol-associated cirrhosis and HCC: trends, projections and risk factors. Nat Rev Gastroenterol Hepatol. 2023;20(1):37–49. 36258033 10.1038/s41575-022-00688-6 PMC9579565

[6] Xiang D et al. Oncofetal MCB1 is a functional biomarker for HCC Personalized Therapy. Adv Sci (Weinh), 2024: p. e2401228. 10.1002/advs.202401228 PMC11615823 39402741

[7] Xiang DM, et al. Oncofetal HLF transactivates c-Jun to promote hepatocellular carcinoma development and sorafenib resistance. Gut. 2019;68(10):1858–71. 31118247 10.1136/gutjnl-2018-317440

[8] Tohyama O, et al. Antitumor activity of lenvatinib (e7080): an angiogenesis inhibitor that targets multiple receptor tyrosine kinases in preclinical human thyroid cancer models. J Thyroid Res. 2014;2014:p638747. 10.1155/2014/638747 PMC4177084 25295214

[9] de Ruiz M, et al. beta-catenin activation promotes Immune escape and resistance to Anti-PD-1 therapy in Hepatocellular Carcinoma. Cancer Discov. 2019;9(8):1124–41. 31186238 10.1158/2159-8290.CD-19-0074 PMC6677618

[10] Bartel DP. MicroRNAs: genomics, biogenesis, mechanism, and function. Cell. 2004;116(2):281–97. 14744438 10.1016/s0092-8674(04)00045-5

[11] Lee H, et al. Biogenesis and regulation of the let-7 miRNAs and their functional implications. Protein Cell. 2016;7(2):100–13. 26399619 10.1007/s13238-015-0212-y PMC4742387

[12] Ambros V. The functions of animal microRNAs. Nature. 2004;431(7006):350–5. 15372042 10.1038/nature02871

[13] Calin GA, Croce CM. MicroRNA signatures in human cancers. Nat Rev Cancer. 2006;6(11):857–66. 17060945 10.1038/nrc1997

[14] Zhang B, et al. microRNAs as oncogenes and tumor suppressors. Dev Biol. 2007;302(1):1–12. 16989803 10.1016/j.ydbio.2006.08.028

[15] DeSano JT, Xu L. MicroRNA regulation of cancer stem cells and therapeutic implications. AAPS J. 2009;11(4):682–92. 19842044 10.1208/s12248-009-9147-7 PMC2782081

[16] Croce CM. Causes and consequences of microRNA dysregulation in cancer. Nat Rev Genet. 2009;10(10):704–14. 19763153 10.1038/nrg2634 PMC3467096

[17] Han T, et al. Downregulation of MUC15 by miR-183-5p.1 promotes liver tumor-initiating cells properties and tumorigenesis via regulating c-MET/PI3K/AKT/SOX2 axis. Cell Death Dis. 2022;13(3):200. 35236826 10.1038/s41419-022-04652-9 PMC8891362

[18] Lindor NM, et al. Germline miRNA DNA variants and the risk of colorectal cancer by subtype. Genes Chromosomes Cancer. 2017;56(3):177–84. 27636879 10.1002/gcc.22420 PMC5245119

[19] He H, et al. MicroRNA-3191 promotes migration and invasion by downregulating TGFBR2 in colorectal cancer. J Biochem Mol Toxicol. 2019;33(6):e22308. 30770602 10.1002/jbt.22308

[20] Li H, et al. HLF regulates ferroptosis, development and chemoresistance of triple-negative breast cancer by activating tumor cell-macrophage crosstalk. J Hematol Oncol. 2022;15(1):2. 34991659 10.1186/s13045-021-01223-x PMC8740349

[21] Zhou T, et al. m6A RNA methylation-mediated HNF3gamma reduction renders hepatocellular carcinoma dedifferentiation and sorafenib resistance. Signal Transduct Target Ther. 2020;5(1):296. 33361765 10.1038/s41392-020-00299-0 PMC7762754

[22] Shepelev MV, Korobko IV. Pak6 protein kinase is a novel effector of an atypical rho family GTPase Chp/RhoV. Biochem (Mosc). 2012;77(1):26–32. 10.1134/S0006297912010038 22339630

[23] Chen XD, Zhao W, Shen AG. Expression and role of PAK6 after spinal cord injury in adult rat. Chin J Traumatol. 2011;14(5):277–81. 22118481

[24] Durkin CH, et al. RhoD inhibits RhoC-ROCK-Dependent cell contraction via PAK6. Dev Cell. 2017;41(3):315–e3297. 28486133 10.1016/j.devcel.2017.04.010 PMC5425256

[25] Yang Q, et al. PAK6 promotes cervical cancer progression through activation of the Wnt/beta-catenin signaling pathway. Oncol Lett. 2020;20(3):2387–95. 32782556 10.3892/ol.2020.11797 PMC7400107

[26] Li T, et al. Mitochondrial PAK6 inhibits prostate cancer cell apoptosis via the PAK6-SIRT4-ANT2 complex. Theranostics. 2020;10(6):2571–86. 32194820 10.7150/thno.42874 PMC7052886

[27] Zheng J, et al. p21-activated kinase 6 controls mitosis and hepatocellular carcinoma progression by regulating Eg5. Biochim Biophys Acta Mol Cell Res. 2021;1868(2):118888. 33098954 10.1016/j.bbamcr.2020.118888

[28] Chen H, et al. Expression and prognostic significance of p21-activated kinase 6 in hepatocellular carcinoma. J Surg Res. 2014;189(1):81–8. 24576777 10.1016/j.jss.2014.01.049

[29] Liu W, et al. Tumor suppressive function of p21-activated kinase 6 in Hepatocellular Carcinoma. J Biol Chem. 2015;290(47):28489–501. 26442588 10.1074/jbc.M115.658237 PMC4653705

