# Supplementary material for: miR-3191 promotes the proliferation and metastasis of hepatocellular carcinoma via regulating PAK6
Source: Infect Agent Cancer. 2024 Dec 18;19:64. doi: 10.1186/s13027-024-00628-w (PMC11654304; doi:10.1186/s13027-024-00628-w)

**miR-3191** **Promotes the Proliferation and Metastasis of Hepatocellular Carcinoma via Regulating PAK6**

Anqi Xie^1,#^, Hengjie Wang^2,#^, Jingchen Huang^3,#^, Minmin Sun^4,*^ Lin Chen^1,*^

^1^Community Health Service Center, Zhongshan Street, Songjiang District, Shanghai, China;

^2^Department of Hepatic Surgery, Kunshan Hospital of Traditional Chinese Medicine. Kunshan, Jiangsu Province, 215300, China;

^3^The National University of Malaysia, Kuala Lumpur, Malaysia;

^4^Department of Hepatic Surgery, Third Affiliated Hospital of Second Military Medical University, Shanghai, 200438, China;

^#^These authors contributed equally to this work.

***Corresponding authors.**

Address: Department of Hepatic Surgery, Third Affiliated Hospital of Second Military Medical University, 225 Changhai Road, 200438 Shanghai, China. Tel.: +86 2181875366. E-mail addresses: sunminmin_ehbh@126.com (Minmin Sun), lynnchan1001@163.com (Lin Chen).

**Running title:** miR-3191 drives HCC cells progression

**Supplementary Tables**

**Supplementary Table 1. Clinicopathological features of 180 HCC patients in Cohort 1**

| Characteristics |  |  | miR-3191 low (n=90) | miR-3191 high (n=90) | p value |
| --- | --- | --- | --- | --- | --- |
| Age(year) | ≤50 |  | 50 | 52 | >0.05 |
|  | >50 | | 40 | 38 |  |
| Gender | Male | | 72 | 69 | >0.05 |
|  | Female | | 18 | 21 |  |
| HBsAg | Positive | | 62 | 81 | <0.05 |
|  | Negative | | 28 | 9 |  |
| AFP(μg/L) | ≤400 |  | 54 | 30 | <0.05 |
|  | >400 |  | 36 | 60 |  |
| Tumor size(cm) | ≤5 | | 43 | 25 | <0.05 |
|  | >5 | | 47 | 65 |  |
| Tumor number | Single |  | 70 | 73 | >0.05 |
|  | Multiple |  | 20 | 17 |  |
| Portal vein tumor thrombus | Yes |  | 12 | 32 | <0.05 |
|  | No |  | 78 | 58 |  |
| Pathological satellite | Yes |  | 41 | 46 | >0.05 |
|  | No |  | 49 | 44 |  |
| BCLC stage | A |  | 57 | 54 | >0.05 |
|  | B or C |  | 22 | 24 |  |
| TNM | I-II |  | 72 | 53 | <0.05 |
|  | III-IV |  | 18 | 37 |  |

HBsAg, hepatitis B virus surface antigen;

AFP, α-fetoprotein;

TNM, Tumor-Nodes-Metastasis;

BCLC, Barcelona Clinic Liver Cancer Staging.

**Supplementary Table 2. Clinicopathological features of 113 HCC patients in Cohort 2**

| Characteristics |  |  | miR-3191 low (n=57) | miR-3191 high (n=56) | p value |
| --- | --- | --- | --- | --- | --- |
| Age(year) | ≤50 |  | 30 | 32 | >0.05 |
|  | >50 | | 27 | 24 |  |
| Gender | Male | | 46 | 45 | >0.05 |
|  | Female | | 11 | 11 |  |
| HBsAg | Positive | | 39 | 50 | <0.05 |
|  | Negative | | 18 | 6 |  |
| AFP(μg/L) | ≤400 |  | 31 | 17 | <0.05 |
|  | >400 |  | 26 | 39 |  |
| Tumor size(cm) | ≤5 | | 27 | 14 | <0.05 |
|  | >5 | | 30 | 42 |  |
| Tumor number | Single |  | 45 | 42 | >0.05 |
|  | Multiple |  | 12 | 14 |  |
| Portal vein tumor thrombus | Yes |  | 5 | 17 | <0.05 |
|  | No |  | 52 | 39 |  |
| Pathological satellite | Yes |  | 23 | 25 | >0.05 |
|  | No |  | 34 | 31 |  |
| BCLC stage | A |  | 40 | 26 | <0.05 |
|  | B or C |  | 17 | 30 |  |
| TNM | I-II |  | 47 | 43 | >0.05 |
|  | III-IV |  | 10 | 13 |  |

HBsAg, hepatitis B virus surface antigen;

AFP, α-fetoprotein;

TNM, Tumor-Nodes-Metastasis;

BCLC, Barcelona Clinic Liver Cancer Staging.


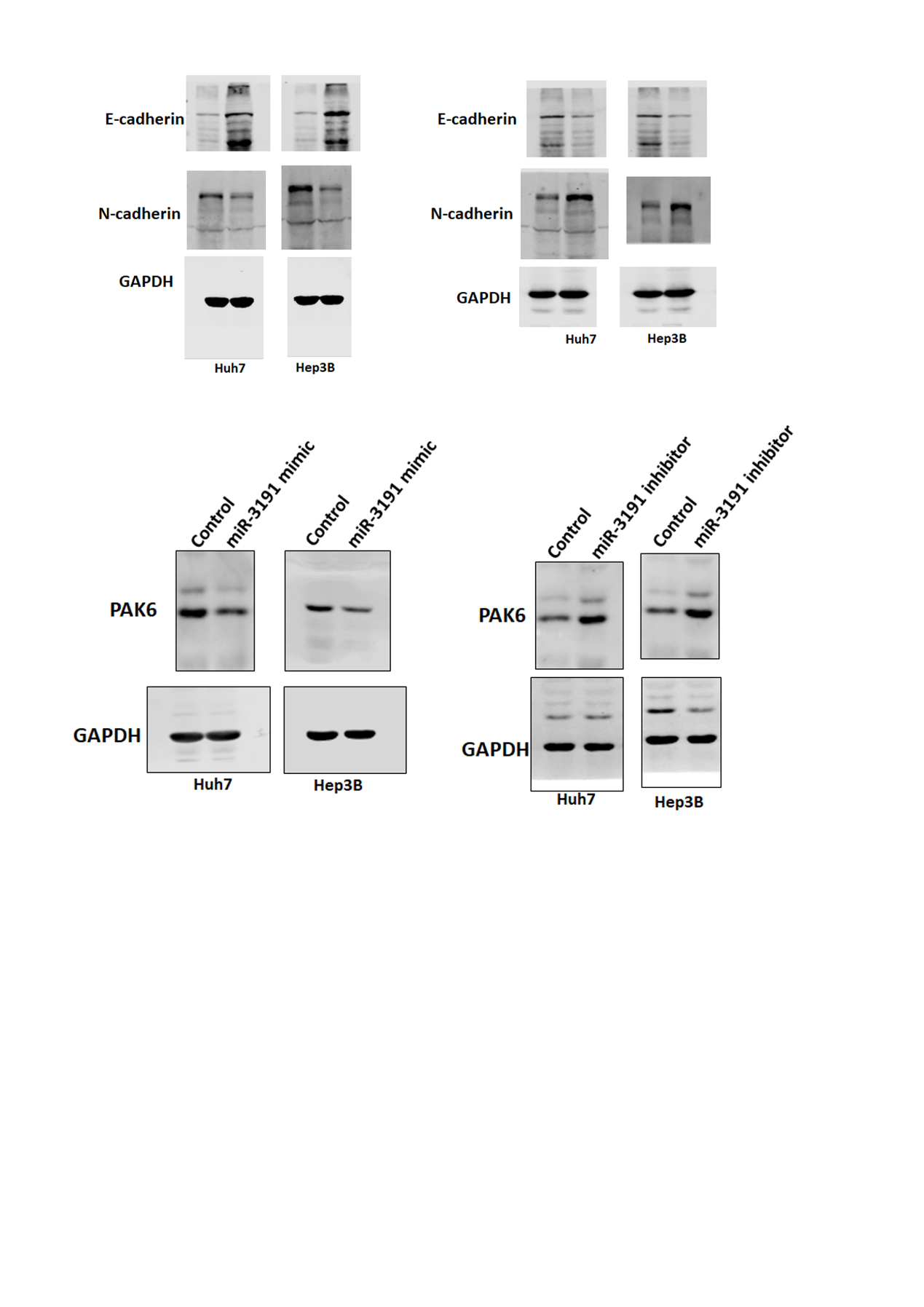

Supplement: Supplementary file 1 — Supplementary Material 1 [file 13027_2024_628_MOESM1_ESM.docx]
